# Functions and regulatory mechanisms of resting hematopoietic stem cells: a promising targeted therapeutic strategy

**DOI:** 10.1186/s13287-023-03316-5

**Published:** 2023-04-11

**Authors:** Xinyu Tang, Zhenzhen Wang, Jingyi Wang, Siyuan Cui, Ruirong Xu, Yan Wang

**Affiliations:** 1 Shandong University of Traditional Chinese Medicine, Jinan, China; 2 Department of Hematology, Affiliated Hospital of Shandong University of Traditional Chinese Medicine, No. 16369 Jingshi Road, Lixia District, Jinan, 250014 China; 3 Institute of Hematology, Shandong University of Traditional Chinese Medicine, Jinan, China; 4 Shandong Provincial Health Commission Key Laboratory of Hematology of Integrated Traditional Chinese and Western Medicine, Affiliated Hospital of Shandong University of Traditional Chinese Medicine, Jinan, China

**Keywords:** Hematopoietic stem cells, Resting, Self-replication, Differentiation, Regulatory mechanism, Hemopathy

## Abstract

Hematopoietic stem cells (HSCs) are the common and essential precursors of all blood cells, including immune cells, and they are responsible for the lifelong maintenance and damage repair of blood tissue homeostasis. The vast majority (> 95%) of HSCs are in a resting state under physiological conditions and are only activated to play a functional role under stress conditions. This resting state affects their long-term survival and is also closely related to the lifelong maintenance of hematopoietic function; however, abnormal changes may also be an important factor leading to the decline of immune function in the body and the occurrence of diseases in various systems. While the importance of resting HSCs has attracted increasing research attention, our current understanding of this topic remains insufficient, and the direction of clinical targeted treatments is unclear. Here, we describe the functions of HSCs, analyze the regulatory mechanisms that affect their resting state, and discuss the relationship between resting HSCs and different diseases, with a view to providing guidance for the future clinical implementation of related targeted treatments.

## Background

**Fig. 1:**
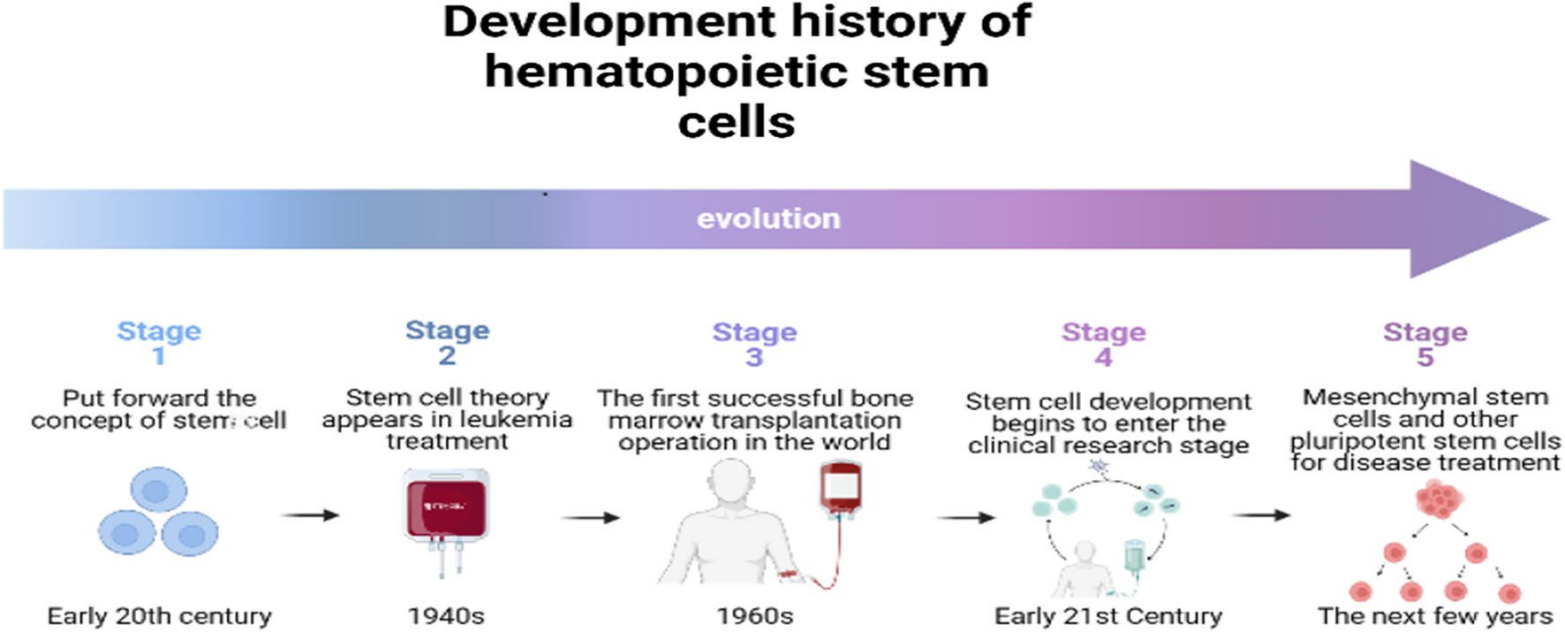
Development history of stem cells. The “stem cell theory” originated in the twentieth century and was gradually developed and then utilized in clinical treatments. The efficacy of bone marrow hematopoietic stem cell transplantation in the treatment of hematological diseases has now been widely recognized. In addition, the functions of mesenchymal stem cells, embryonic stem cells, induced pluripotent stem cells, and other stem cells with strong differentiation abilities are now also being studied and used in clinical treatments. (It is original by the author, the components in this figures were created with BioRender.com)

Hematopoietic stem cells (HSCs) are adult stem cells that have the capacity for long-term self-renewal and can differentiate into various mature blood cells [1]. HSC research (see Fig. 1) began at the start of the twentieth century. When Maximow proposed that human cells develop from one cell or another. However, as there was no evidence to support in-depth research, of this topic, the conjecture did not attract any further attention at the time [2]. By the 1940s, the treatment of leukemia had become a focus of medical. However, when this research reached an impasse, the “stem cell theory” was once again proposed. Although many people have questioned it, research on stem cells has still entered people's vision [3]. After more than 20 years of research, the world's first bone marrow (BM) transplantation operation was successfully completed in 1968, in Washington, D.C., USA. Improved therapeutic effects were achieved by injecting HSCs that had been successfully matched to leukemia patients through intravenous injection, and this promoted the rapid development of HSC research. Stem cell transplantation remains the most effective method to treat leukemia and other blood diseases [4]. Based on the success of this operation, the development prospects of stem cells received considerable attention, and since 1990, stem cell research has been widely supported around the world.

Initially, stem cell research involved the selection of HSCs from BM. However, it is now understood that the material downstream of HSCs in the BM can differentiate into a variety of cell types, and mesenchymal stem cells (MSCs) and embryonic stem cells (ESCs) are gradually becoming better understood. At the beginning of the twenty-first century, stem cell technology began to be used in clinical research with a variety of illnesses such as immune system diseases, nerve injury, and diabetes [5]. A few years later, scientists discovered induced pluripotent stem cells, and these were then increasingly used in clinical disease treatments. Stem cells have since continued to be a focus of regenerative medicine and hematological system disease research. Among these fields, hematopoietic reconstitution defects such as damaged homing and BM-biased differentiation are hot topics [6]. Due to this work, the functional activities of HSCs are being increasingly recognized, and several pluripotent stem cells downstream of HSCs are now used in clinical treatments. In the future, it is expected that HSCs will play an increasingly important role in clinical treatments, in conjunction with new technologies (such as cell reprogramming).

All peripheral blood cells differentiate from the HSC pools in the BM [7], and the production of mature cells by HSCs is a multi-step process in which the differentiation potential decreases [8]. Under physiological conditions, most HSCs, especially long-term hematopoietic stem cells (LT-HSCs) that are located upstream, exist in a resting state and only become activated when the body is stimulated by stress [9]. Importantly, they can be restored to their resting state when they are no longer needed [10], and this is conducive to the long-term survival of HSCs and helps to avoid depletions and thus the maintenance of lifelong hematopoietic function. Furthermore, it also helps to ensure that HSCs can immediately cope with blood loss, BM transplantation mobilization, and other conditions and special needs of the body.

Distinguishing the resting state of HSCs is an ongoing aim of researchers. Flow cytometry can be used to analyze the expression of cell surface antigen markers and related intracellular factors in HSCs [11], and this strategy has been used for HSC isolation in mice [12]. The HSCs isolated from mice were purified using the cell surface marker Lin-c-Kit + Sca1 + (LKS+) [13], and a subset of the HSCs with the phenotype Lin (−) Sca (+) kit (+) CD38 (+) CD34 (−) was isolated from 5-color fluorescent-activated cells [14]. The surface markers of Flk2/Flt3, CD48, CD34, and CD150 were also used to distinguish LT-HSCs from short-term HSCs (ST HSCs) [15, 16]. Thy-1 low Sca-1 + Lineage-c-kit + cells in aging mice reportedly include CD48 + and/or CD150 cells, which are unable to perform recombination [17]. There is also a significant difference in the self-renewal and differentiation of HSCs in Sca-1+ and Sca-1- [18], and this research provides a method to test the active state of HSCs. Cell sorting is indispensable when studying the functional activities of HSCs, and technological progress has made it possible to develop targeted therapies.

The activation and resting status of HSCs is regulated by complex mechanisms and is related to the occurrence, development, and treatment prognosis of each disease [19]. Heterogeneous and diversified HSC abnormalities exist in many diseases, and HSC transplantation is an important strategy in clinical treatments. This method depends on the functional characteristics of the resting HSCs that are used to rebuild the blood. However, our limited understanding of resting HSCs hinders the development of clinical disease treatments that can target them.

In this review, we briefly summarize the functions of HSCs, analyze their molecular regulatory mechanisms when resting and discuss the correlation between resting HSCs and the occurrence and development of different diseases. The overall aim is to provide a theoretical foundation for future research on resting HSCs and identify potentially feasible strategies for targeted clinical therapies.

## Functions of HSCs

### Self-renewal and replication

Self-renewal refers to the process by which stem cells generate at least one daughter cell that can retain stem cell characteristics during division. This process enables a stem cell bank to exist throughout a life cycle and thus is basis for the maintenance of a lifelong regenerative potential [20]. The maintenance of the normal self-renewal function of HSCs is related to their asymmetric mitosis [21], that is, one of the two daughter cells can be an early progenitor cell, while the other retains all the stem cell characteristics. This means that the number of HSCs remains unchanged regardless of how many times they divide, and this enables them to constantly generate progenitor cells to meet an organism’s differentiation needs [22, 23].

Most HSCs exist in a resting state (G0 phase) in the body and do not undergo DNA synthesis or mitosis [24]. Wilson et al. identified resting mouse HSCs using flow cytometry and label retention analysis, and the results showed that the HSCs split approximately once every 145 days, or five times in their lifetime, and the entire HSC pool would flip once every period of time [25]; that is, HSCs did not enter and exit the cell cycle randomly, but changed reversibly from a dormant state to an active self-renewal state under specific conditions.

Although there is a powerful resting bank of HSCs in the body, they can still be effectively activated to achieve self-renewal, rapid expansion, and differentiation in response to BM injury or stress stimulation [26]. When the body reaches normal homeostasis, the activated HSCs resume their resting state and more than 95% will remain in the G0 phase [27]. Resting HSCs are beneficial for the repair of slight gene mutations that occur during mitosis and help to avoid the development of irreversible multi-gene mutations that may cause malignant transformations in stem cells. When the body experiences hypoxia, infection, blood loss, trauma, and other stress reactions, the number of HSCs that change from a resting to a proliferative state increases, to meet the body's hematopoietic needs [28]; however, the HSCs still undergo asymmetric mitosis to maintain the characteristics of self-renewal and self -maintenance.

When HSC self-renewal and self-maintenance is abnormal, symmetrical mitosis occurs and all daughter cells will differentiate into progenitor cells after division. The number of HSCs is thus continuously reduced and gradually depleted, and this is followed by the disappearance of early progenitor cells, which eventually leads to hematological failure. Alternatively, one HSC can divide into two during symmetrical mitosis, resulting in continuous amplification [29]. The resting state of HSCs will affect their self-renewal ability, normal activation, and resting characteristics, which all significantly impact the maintenance of lifelong hematopoietic functions in the body.

### Multi-lineage differentiation

**Fig. 2:**
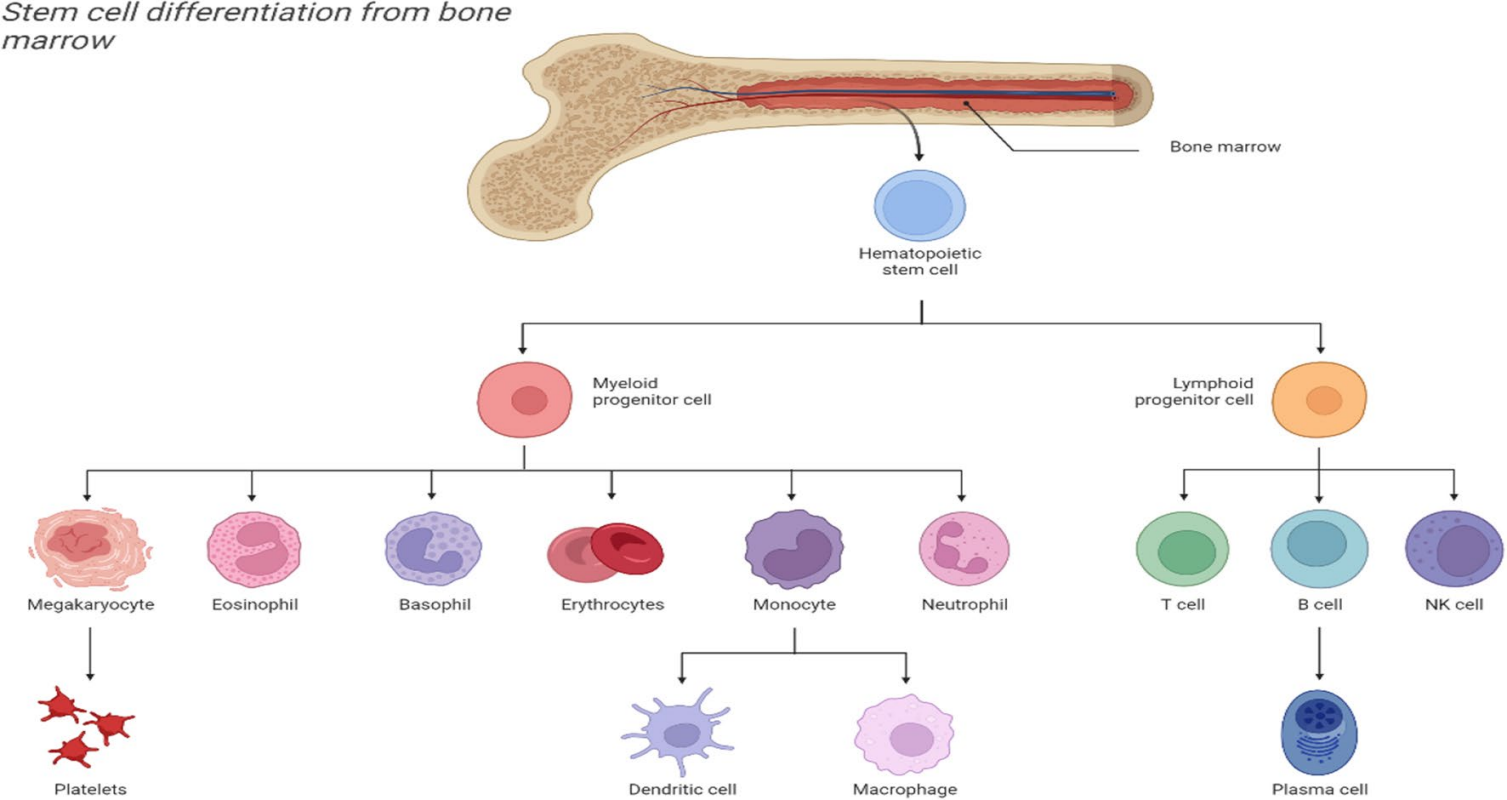
Multi-lineage differentiation of bone marrow hematopoietic stem cells. Hematopoietic stem cells in the bone marrow differentiate into myeloid progenitor and lymphoid progenitor cells, and the lymphoid progenitor cells further differentiate into T cells, B cells, and NK cells, among which the B cells can differentiate into plasma cells. Myeloid progenitor cells further differentiate into megakaryocytes, eosinophils, basophils, erythrocytes, monocytes, and neutrophils, among which the monocytes include DC cells and macrophages. (It is original by the author, the components in this figures were created with BioRender.com)

HSCs are the primary source of hematopoietic differentiation. The HSC pools include three main cell types: LT-HSCs, ST HSCs, and multipotent progenitor cells (MPPs) [30], with varying differentiation potentials. LT-HSCs are at the top of the hematopoietic cascade, and they can maintain multi-lineage hematopoietic reconstruction for a long period; notably, they are the only cell population with true HSC characteristics. The ST HSCs and progenitor cells can only maintain normal hematopoiesis for 6–8 weeks [31]. HSCs undergo asymmetric division to produce daughter cells and further proliferate and differentiate into a complete hematopoietic lineage [32], which mainly differentiates into two branches of the lymphoid lineage and the myeloid lineage, namely the common lymphoid progenitor cells and the common myeloid progenitor cells. Finally, peripheral effector cells are established in each lineage, including T cells, B cells, and NK cells in the lymphoid lineage, as well as red blood cells, platelets, granulocytes, macrophages, and dendritic cells [32, 33] (see Fig. 2). The life span of these terminally differentiated blood cells is usually short, and the HSCs need to continuously differentiate to compensate for daily losses.

The differentiation of HSCs is related to the aging of organisms, and the occurrence of various hematopoietic system diseases is often accompanied by abnormal HSC differentiation. According to the different differentiation abilities, HSCs can be divided into lymphatic biased hematopoietic stem cells (Ly Bi HSCs), myeloid-biased hematopoietic stem cells (My Bi HSCs), and balanced hematopoietic stem cells, during cell aging, and HSCs appear to transfer from Ly Bi HSCs to My Bi HSCs [34, 35]. This abnormal, cellular autonomy bias leads to hematopoietic dysfunction, imbalance in the proportion of lymphocytes and myeloid cells, and damage to the immune response of the body under stress, which further promotes aging [31].

A study found that the number of HSCs increased in an aging body when compared to that in a younger body [36]. Animal experiments also show that when compared with young mice, the proportion of myeloid cells in the peripheral blood of old mice is significantly higher, and this includes Mac1+ and Gr1+ hematopoietic cells [37]. This may be because the proportion of HSCs in the BM of old mice is relatively high [38], but their function is defective, and they cannot maintain normal hematopoiesis [39]. After BM transplantation, resting HSCs in the body are activated in the cell cycle and differentiate into different hematopoietic lineages to rebuild the hematopoietic system [40]. However, after transplanting senescent HSCs into young mice, the young mice were found to show “senescence” [41, 42].

Abnormal differentiation of HSCs is associated with aging-related diseases of the BM. The factors that affect the differentiation of HSCs should be studied further as this will help to facilitate the development of targeted treatments for disease.

## Regulatory mechanisms of resting HSCs

**Fig. 3:**
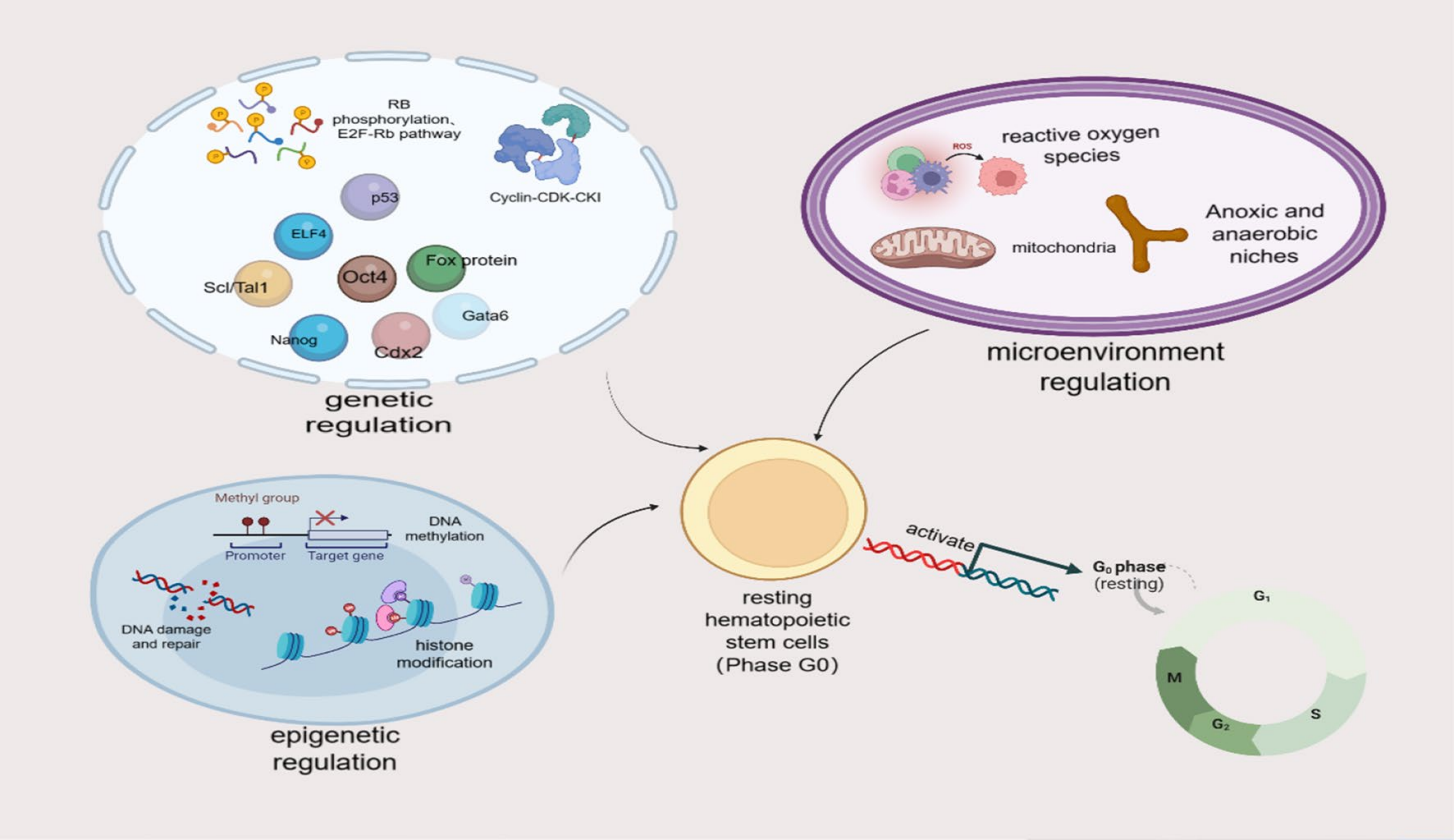
Multiple mechanisms are involved in regulating the resting and activation of HSCs. The mechanisms regulating resting HSCs predominately include genetic, epigenetic, and microenvironment regulations. Genetic regulations include cyclin, cyclin-dependent kinase (CDK), cyclin-dependent kinase inhibitor (CKI), cell cycle regulation, transcription factor regulation, RB phosphorylation, and E2F Rb pathway regulation, and ubiquitination regulation. Epigenetic regulations include histone modification regulation, DNA methylation regulation, DNA damage and repair regulation. Microenvironment regulations include active oxygen regulation and anoxic and anaerobic niche regulation. (It is original by the author, the components in this figures were created with BioRender.com)

The quiescence and activation of HSCs is an active and reversible process, and this ability to enter and exit the cell cycle at any time is conducive to the normal function of HSCs and a timely response to the various stress needs of the body [43]. This process is strictly regulated by various mechanisms both inside and outside of cells (see Fig. 3), including genetic, epigenetic, and hematopoietic microenvironment regulations. In addition, HSCs can also return to a resting state (homing), which is a prerequisite for their long-term existence in the BM microenvironment.

### Genetic regulation

HSC genetic regulation refers to regulation by the related proteins, transcription factors, and signal transducers involved in the cell cycle. The factors involved in cell cycle regulation mainly include cyclin, cyclin-dependent kinase (CDK), cyclin-dependent kinase inhibitor (CKI), RB protein, E2F-DP1 transcription factor, enzymes that regulate the phosphorylation and dephosphorylation of CDK, and enzymes that make proteins ubiquitin. The complex network of their interactions harmoniously regulates the cell cycle, cell proliferation, growth, and repair; tumor occurrence, development, and metastasis are closely related to the cell cycle [44]. The expression and activity of regulatory cytokines have implications for the clinical treatment of diseases, based on the influence of these factors on the hematopoietic microenvironment. When an abnormal situation occurs in an organism, HSCs can be dynamically regulated via the expression of multiple factors, to promote damage repair in the body, and the treatment of disease [19].

#### Cyclin, CDK, and CKI network regulation

Cyclin can be divided into A, B, D, E, and other types; of these, cyclin D is mainly expressed in the G1 phase [45]. The entry of HSCs from the G0 phase into the G1 phase (exiting the resting state) and from the G1 phase into the S phase, respectively, depends on the activation of CDK4/6 and CDK2; CDK4/6 is a common downstream target of multiple signaling pathways, and it can be combined with cyclin D to form a complex, drive cells to develop from the G1 to S phases and cause cell proliferation, which eventually leads to uncontrolled proliferation [46]. Interestingly, CDK2 mainly binds to cyclin E [47].

Laurenti et al. found that under normal circumstances, LT-HSCs lack CDK6; while short-term hematopoietic stem cells are also in a resting state, they contain high levels of CDK6, allowing rapid entry into the cell cycle after stimulation. Furthermore, the time required for LT-HSCs to exit the resting state is shortened after the forced expression of CDK6 [48].

CKIs mainly include the CDK4/6 inhibitor family (p15, p16, p18, and p19) and the cytokine-induced protein/kinase (CIP/KIP) protein family (p21, p27, and p57 proteins) [49, 50]. At present, the role of CDK4/6 inhibitors in the regulation of HSCs is still unclear, while the role of CIP/KIP subfamily members is relatively clear. CIP/KIP inhibits the activity of the cyclin-CDK complex, reduces the release of E2F, inhibits HSCs to enter the cell cycle, and helps HSCs maintain a resting state [51]. Among them, p21 and p57 have similar effects, knockouts can lead to HSCs depletion [52], and p57 may play a more dominant role in the rest of HSCs [53].

#### Transcription factor regulation

Transcription factors are involved in the regulation of HSC resting, and activation as well as in the proliferation, self-renewal, and differentiation or apoptosis after activation, transcription factors that are closely related to HSCs quiescence mainly include p53, ELF4, Scl/Tal1, the Fox protein family, Oct4, Nanog, Cdx2, and Gata6 [54, 55]. p53 can bind to the promoter region of p21 and activate p21 gene transcription; when cellular DNA is damaged, p53 induces the upregulates downstream p21 expression, inhibits the activity of CDK, and prevents cells from entering the S phase from the G1 phase. Furthermore, the deletion of p53 leads HSCs to enter the cell cycle [56] and promotes the symmetrical division of HSCs to expand the HSC pool [57]; however, the HSCs produced by division are defective and prone to cause neoplastic diseases [58].

The expression of ELF4 is negatively correlated with the maintenance of resting HSCs, and the knockdown of ELF4 can lead to an increase in the number of resting HSCs [59]. Scl/Tal1 can positively maintain the resting state of HSCs and inhibit their transition from the G0 to G1 phases. The Scl/Tal1 was notably found to be highly expressed in LT-HSCs [60].

Hou et al. found that Foxm1 was highly expressed in LT-HSCs. Its downregulation reduces human resting CD34 + HSCs, promotes the proliferation of HSCs, but does not affect their differentiation [61].

Adrenaline released from nerve endings in the BM can interact with the β-3 adrenaline receptor (β3-AR) binding, causing the downregulation of transcription factors Sp1 and CXCL12; this regulates the activation of HSCs [62].

Several transcription factors such as Oct4, Gata6, Nanog, and Cdx2 also have important roles in the process of stem cell self-renewal and differentiation, which can not only maintain the activity of stem cells, but also inhibit the expression of genes that promote differentiation, leading to lineage switching between stem cells [63]. Multiple molecular mechanisms work together to regulate the HSC resting state.

#### RB phosphorylation and E2F-Rb pathway regulation

As a tumor suppressor, the Rb protein can prevent cells from entering the S phase, inhibit cell growth, and play an important role in the negative regulation of the cell cycle and tumor progression. E2F is a transcription factor necessary for the promotion of cyclin and CDK protein transcription in the S phase, and the Rb protein inhibits the gene transcription required to move from the G1 to S phase by binding with E2F [64]. HSCs in the resting state are activated into the G1 phase; the Rb protein is phosphorylated by cyclin-CDK and then releases E2F bound to it, inducing cells to enter the cell cycle. Members of the CDK4/6 inhibitor family can inhibit the binding of cyclin D and CDK4/6, as well as the phosphorylation of Rb proteins, which leads to cell cycle arrest, and has an antitumor role [65]. The simultaneous knockdown of p27 and p57 also stimulates Rb phosphorylation in HSCs, leading to the loss of resting HSCs [53, 66], while the knockdown of p27 alone has no obvious effect.

The fibrous layer related polypeptide 2 alpha (LAP2α), is a nuclear protein that is dynamically associated with chromatin in the cell cycle. It can participate in the regulation of cell cycle progression through the E2F Rb pathway, and LAP2α protein expression is downregulated when exiting the cell cycle [67]. The deletion of LAP2α in mice can promote a proliferative stem cell phenotype in progenitor cells and delay stem cell differentiation [68]. LAP2α has also been found to act as an important regulator of osteogenic differentiation in human adipose stem cells (hASCs) through the regulation of the NF-κB signaling pathway, and knocking down LAP2α leads to impaired osteogenic hASC differentiation [69].

#### Regulation of ubiquitination

In addition, the late cell division promotion complex/cyclin (APC/C) is one of the important E3 ubiquitin ligase complexes, which participates in the strict regulation of the cell cycle. The deletion of the Anapc2 (core subunit of APC/C) allele produces defective APC/C, which causes BM failure and damages the functional activity of HSCs [70]. The sequence-specific single strand DNA binding protein (SSBP) can enhance transcriptional activity by preventing the ubiquitination of LDB1 by E3 ubiquitin ligase RNF12/RLIM [71], and it plays a key role in regulating the expression and function of HSC genes [72]. The deletion of histone H2A ubiquitinase MYSM1 drives HSCs from a resting state to a stress cycle, that is prone to apoptosis, leading to the depletion of the HSCs library during the stress response. These data confirm the important role of MYSM1 in maintaining the resting state and survival of HSCs [73].

The above evidence indicates that genetic regulation can affect the cell cycle progression of HSCs, which is essential for HSCs to maintain their resting potential and perform their normal functions; specific changes in genetic regulation may cause disease. Transcription factors and phosphorylation are important factors for clinical treatment as they can change the action pathway of cytokine or signaling pathways [63].

### Epigenetic regulation

The tight regulation of gene expression is more important for HSCs than for terminally differentiated cells, as dysregulated gene expression can exist in HSCs for a long period due to their lifelong capacity for self-renewal [74]. Furthermore, the multi-lineage differentiation of HSCs involves the coordinated regulation of multiple gene expression programs [75, 76]. In addition to transcription factors, gene expression controlling cell differentiation also involves a variety of epigenetic changes [77, 78], and lineage-related genes in HSCs may have specific chromatin structures and epigenetic modifications [79, 80]. The epigenetic mechanisms regulating the expression of lineage-related genes can help prepare for HSC multi-lineage differentiation [81–83], or may lead to changes in the chromatin structures and functions of gene sites [84, 85].

Epigenetic regulation is a key factor controlling the functions of HSCs and regulating their aging processes as well as determining cell fate. The main methods of this mechanism are histone modification and DNA methylation, and post-translational modifications of histones include including acetylation, methylation ubiquitination, and ubiquitination [86]. In blood system diseases, the occurrence of diseases is often accompanied by the abnormal expression of some genes. Epigenetic regulation can regulate the pedigree of the relevant gene, maintain stability at the gene level, and play a role in disease treatment. This phenomenon is also applicable to other systemic diseases [84].

#### Regulation of histone modifications

Joanne et al. found that histone and DNA modifications are associated with the enhanced transcription of related genes in HSCs and MPPs and that specific combinations of epigenetic modifications can mediate the transcription of lineage-related genes in HSCs [87]. Analysis of the chromatin structure of lineage-related genes in early hematopoiesis showed that the chromatin structure is important for maintaining the functional activity of HSCs. For example, knocking out the chromatin-binding protein sin3 destroys the resting state of HSCs and impairs their ability to differentiate and self-renew [88]. The histone acetyltransferase HBO1 can maintain the remaining HSCs in the process of hematopoiesis, and after HBO1 is lost, the resting HSCs are activated into the cell cycle, producing progenitor cells at the expense of self-renewal, eventually leading to a depletion in available HSCs [89].

#### DNA methylation regulation

In addition to histone modifications, DNA methylation is also one of the epigenetic regulation that affects the resting state and function of HSCs. EZH2 can inhibit gene transcription by participating in DNA methylation, and its abnormal expression can lead to a significant increase in HSCs [90]. While knockout of DNA methyltransferase 3 (DNMT3) can promote the self-replication of HSCs and inhibit their differentiation [91], on this basis, this change is more significant after the simultaneous knockout of DNMT3b [92], which indicates that DNMT3a and DNMT3b have synergistic effects and jointly participate in the regulation of HSCs. The TET2 gene, which promotes DNA demethylation, also participates in the regulation of HSC function, and the destruction of TET2 can induce the amplification of HSCs and MPP, leading to the accumulation of precancerous clones [93]. DNA methylation of paternal or maternal alleles is crucial for embryonic HSCs [94]. Venkatraman et al. [95] found that DNA methylation from maternal lines was an important factor that helped LT-HSCs to maintain a resting state, and knocking out the differential methylated region upstream of maternal H19 could disturb the resting state and damage their function.

#### DNA damage and repair regulation

The DNA damage response (DDR) plays an important role in the epigenetic regulation of HSCs. Aging HSCs exhibit DNA damage accumulation and abnormal epigenetic landscape in histones and DNA [96]; these aging HSCs are also related to inflammatory reactions in the body. HSCs are key participants in the systemic inflammatory response, which can integrate the inflammatory response signal into the cellular response, and establish an inflammatory adaptation signal axis between the BM hematopoietic response and peripheral stress [97]. Infection and inflammatory diseases often promote the aging of HSCs, reduce their function, and lead to the damage of BM hematopoietic function and the reduction of cell regeneration ability. Long-term inflammation can also cause BM niche cells to produce secondary inflammatory signals through direct and indirect effects, further affecting the functional activity of HSCs [98]. Inflammation is clearly the driving factor of BM somatic cell evolution, and the function of HSCs is closely affected by inflammation, which leads to aging of HSCs and tumor occurrence.

A study involving a DNA repair defect mouse model confirmed that DDR in cells is closely related to HSC function and plays an important role in the tissue homeostasis of the hematopoietic system [31]. DDR can induce chromatin remodeling and epigenetic modifications, causing a variety of cellular reactions, including DNA repair, cell cycle regulation, cell aging, and death [99–101]. DNA damage activates damage monitoring points, and p53 is the core of the G1 damage checkpoint. Downstream of the p53 signaling pathway, the DNA damage response is regulated through p21 and Puma, which have key roles in responses of HSCs to DNA damage repair to maintain functional stability [102, 103].

Ku70 is one of the main mechanisms involved in DNA repair. Yulan et al. found that the self-renewal of HSCs and the BM hematopoietic niche in Ku70-deficient mice is abnormal, and this defect is related to the absence of resting HSCs. The overexpression of Bcl2 can reduce HSC deficiency in Ku70-deficient mice by restoring the resting state of HSCs [104]. The deletion of DNA repair factors leads to the accumulation of DNA damage and can seriously damage HSC function [105]. Furthermore, mice with DNA repair defects are more likely to age and show hematopoietic function defects [106]. Inappropriate DNA damage repairs may negatively regulate the maintenance of HSCs, damage the function of HSCs, and lead to disease [107].

This evidence indicates that epigenetic regulations are important for resting state regulations of HSCs and the maintenance of their functions.

### Microenvironment regulation

**Fig. 4:**
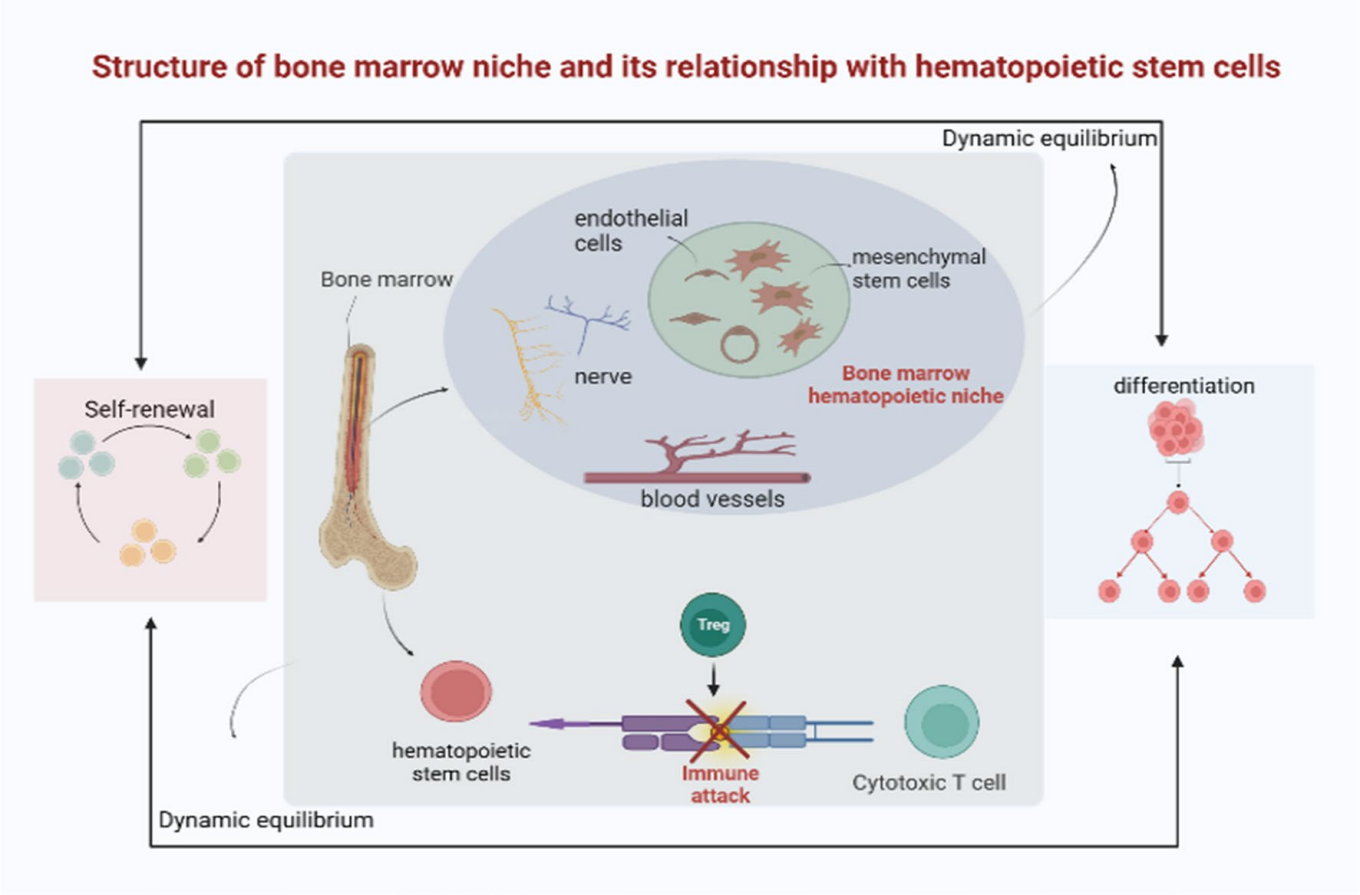
Structure and function of the bone marrow hematopoietic niche. The bone marrow hematopoietic niche is the spatial location and physiological microenvironment for the survival and self-renewal of hematopoietic stem cells, which is mainly composed of blood vessels, nerve, mesenchymal stem cells and endothelial cells. It maintains a dynamic balance between self-renewal and differentiation of stem cells. Regulatory T cells are located in bone marrow and protect hematopoietic stem cells from immune attack. (It is original by the author, the components in this figures were created with BioRender.com)

The functional activity of HSCs is also regulated by external cell signals that are received from their niche, and the interaction between resting HSCs and a niche -specific microenvironment is essential for functional maintenance and the protection of HSC pools from premature failure under various stress stimuli. As the microenvironment in the body changes at different stages of life, the way HSC’s function is maintained also changes accordingly [30]. Understanding the mechanism of changes in the microenvironment will help restore the normal functions of the hematopoietic system and treat disease (see Fig. 4) [108].

#### Reactive oxygen species regulation

In resting HSCs, ROS are maintained at low levels to support their long-term regeneration ability [109]. The HSC pool contains a cell population that can be separated according to the ROS level and redox environment. “Low ROS” populations contain more long-term culture initiating cells (LTC-IC), and the characteristics of these cells help them support long-term hematopoiesis. The reconstruction of hematopoietic systems in patients who lose their hematopoiesis ability after radiotherapy or chemotherapy depends on LTC-IC [110].

The ROS level is a negative regulatory factor for HSCs to maintain a resting state, and high ROS cell populations increase p38 activity, and p16 expression, while decreasing p53 expression [111]. Keisuke found that increased ROS levels activate p38 MAPK and induce HSCs to specifically phosphorylate p38 MAPK, resulting in HSCs that are unable to maintain a resting state; after treatment with antioxidants or p38 MAPK inhibitors, the life of resting HSCs can be prolonged [112]. Similar studies have confirmed that the resting state of HSCs is closely related to low metabolic activity [113]. ROS generated by resting HSCs’ metabolic pathways and replication errors generated during HSCs’ proliferation may damage the integrity of HSCs’ genomes [114].

CD73 and CD150 are important regulators that help maintain HSCs, and the FoxP3+ regulatory T cell population (Treg) has a high expression level of the HSC marker CD150 [115]. Yuichi et al. found that CD73 deletion significantly increased the number of circulating c-Kit + Sca1 + Lin HSCs and CD150 + CD48-c-Kit + Sca1 + Lin- HSCs, as well as the ROS level in HSCs; after treatment with antioxidants, CD73 could reverse the increase in HSCs in mice and protect them from oxidative stress, which helped maintain a resting state [116]. O-linked N-acetylglucosamine transferase (OGT) is highly expressed in HSCs, and its destruction leads to increased ROS levels and apoptosis. HSCs lacking OGT lose their resting state and cannot be maintained in vivo [117].

Superoxide dismutase and catalase can clear ROS in the body, reduce ROS levels, and thus, maintain the long-term self-renewal of HSCs. Further research shows that the overexpression of these two enzymes can reduce ROS levels, promote DNA repair, reduce apoptosis, and significantly promote the long-term survival of HSC transplantations [118]. This shows that the HSC functional defects are related to ROS levels, and antioxidant treatments can restore the reconstruction ability of HSCs and prevent BM failure [119].

#### Anoxic and anaerobic niche regulation

Anoxic and anaerobic environments can also affect the maintenance of resting HSCs. In the adult hematopoietic system, resting HSCs in a hypoxic niche are more sensitive to ROS [120]. In the BM, HSCs are mainly distributed around the endosteal and blood vessels/sinuses, which are anaerobic environments called HSC niches [121], and the energy obtained by anaerobic glycolysis can provide material to help guarantee for the maintenance of resting HSCs [122]. Hypoxia-inducible factor-1 α (HIF-1α) is necessary to maintain the rest of the HSCs and is highly expressed in HSCs [123]. Resting HSCs in HIF-1α-deficient mice were decreased after HSC transplantation, BM suppression, and aging [124].

Uckelmann et al. found that the extracellular matrix adaptor protein Matrilin-4 (Matn4) was one of the negative regulators of stress responses in HSCs, and it is highly expressed in LT-HSCs. In HSCs are stimulated by stress, the expression of Matn4 was found to be down regulated, and the lack of Matn4 led to a stress response and the expansion of HSCs after transplantation [125]. The conditional knockout of CXCL12 can lead to the amplification of HSCs and a reduction in resting LT-HSCs, whereas the knockout of CXCR4, the receptor gene of CXCL12, can cause excessive proliferation of HSCs [126]. Asada et al. found that the inactivation of stem cell factors in leptin receptor (LepR)+ cells leads to the downregulation of HSCs, whereas after the selective removal of CXCL12 from cells expressing LepR, the level of HSCs did not decline, indicating that cells in different niches selectively regulate resting HSCs by secreting specific factors [127]. Hypoxia helps maintain a HSC rest state [128], and the knockout of osteopontin, which is also a negative regulator of HSCs at rest, can increase the number of HSCs in the BM cavity [129].

#### Regulation of adhesion molecules

N-cadherin is a calcium-dependent adhesion molecule that helps regulate the interaction between stem cells and the hematopoietic microenvironment. Arai et al. found that the overexpression of N-cadherin can prevent HSC division, increase the resting storage for HSCs, and maintain their long-term remodeling capacity [130]. N-cadherin may be the downstream target of the Tie2/Ang-1 signaling pathway, which can induce the adhesion of N-cadherin-dependent HSCs [131]. Inactivation of c-Myc can cause an increase in N-cadherin expression on the surface of HSCs, anchor HSCs in the microenvironment [132], and help maintain their resting state.

Very late antigen 4 (VLA-4) is a member of the integrin family of cell adhesion molecules (CAMs), which are expressed in all mononuclear hematopoietic cells. It can combine with fibronectin and the immunoglobulin superfamily molecule VCAM-1, blocking the VLA-4/VACAM-1 pathway and reducing the efficiency of HSCs turning to rest [133].

Genetic, epigenetic, and microenvironment regulations are key factors that affect the entry and exit of HSCs to the resting state. Once the regulatory mechanism is abnormal, the hematopoietic system immediately responds, and the functional activity of resting HSCs changes, resulting in a variety of diseases. In addition, some signal axes are also involved in maintaining resting HSCs, such as the NCoR1/HDAC3-Akt 1/2-mTOR and STAT5B-CD9 axes [134, 135]. The HSC niche, which is the spatial location and physiological microenvironment on which HSCs depend for survival and self-renewal, is closely related to cell regeneration as well as disease treatment, and the regulation of gene expression influences the regulation of HSCs and influences the function of the hematopoietic niche. HSCs are the key to maintaining normal hematopoietic function, and the study of related mechanisms will provide a broad development space for maintaining hematopoietic stability under the conditions of cell stress injury and provide a basis for research into the potential applications of resting HSCs as an intervention target to promote disease treatment.

### Return of resting HSCs

At present, it is possible to detect the homing ability of HSCs in different microenvironments of receptor BM before the initial division of HSCs by transplanting HSCs containing population cells with special markers to recipient animals [136]. The CXCL12/CXCR4 and VCAM1/VLA-4 (integrin α four β 1) signaling pathways were also found to be key pathways that affect HSC homing and stabilize the hematopoietic microenvironment [137]; reducing the sensitivity of the CXCR4 signaling pathway can help activate resting HSCs [138]. Based on the function of CD26 to hydrolyze amino acids at the N-terminus of CXCL12 and block its chemokine -like effect [139], CD26 inhibitors can promote the homing of HSCs after transplantation [140]. As an inhibitor of CD26, tissue factor pathway inhibitor can promote the response of mouse and human HSCs to CXCL12, thus promoting the homing of HSCs [141]. In addition, hyaluronic acid expression on endothelial cells can interact with CD44 on the surface of HSCs to regulate their homing [142]. Osteopontin expression on osteoblasts mainly combines with integrin family molecules on the HSC surface to regulate the homing and maintenance of HSCs [143].

HSC homing may be regulated by different types of the same factor. Adams et al. found that the Ga subunit of the G protein-coupled receptor has two states, activated (as) and inhibited (ai). When CXCL12 in the microenvironment combines with CXCR4 on the surface of HSCs, it releases the ai subunit of the G protein, and this means that ATP cannot catalyze cAMP, thereby inhibiting the activation of HSCs and maintaining the resting state; however, it is unclear whether CXCR4 can also promote the release of as subunits. However, when epinephrine, and prostaglandin E, for example, are combined with their corresponding G protein-coupled receptors, they release the as subunit of the G protein, allowing ATP to be catalyzed to cAMP, and promoting the activation of HSCs [144]. The cAMP content is thus the central regulatory factor determining whether HSCs’ are in a resting or activation stage, and the excavation of its upstream and downstream signaling pathways will further elucidate the regulatory mechanisms controlling the movement of HSCs [145].

HSCs present in the BM can enter the blood, via a process called HSC mobilization, and this is used to harvest a large number of cells for clinical HSC transplantation. At present, G-CSF is the most common clinical mobilization strategy. Although the tolerance was good, the number of HSCs collected was often poor. Researchers are developing new drugs to strengthen mobilization and increase the number of HSCs available for transplantation [146]. At the same time, homing to the BM is required to optimize implanted cells, and homing detection of the HSCs is an important step in assessing their active status and clinical treatment development. However, methods to detect the homings of HSCs are currently limited, and different methods combining imaging techniques to detect the homing ability of HSCs at different time points after transplantation require further exploration.

## Resting HSCs and disease

The self-renewal and multi-directional differentiation of HSCs occurs in both normal and abnormal tissues and cells [147], such as leukemia, which occurs when immature hematopoietic cells in the blood proliferate abnormally [148]. Resting HSCs are usually resistant to conventional chemotherapy and targeted therapy; it is thus crucial that we improve our understanding of these cells to elucidate the relationship between HSCs and disease occurrence and development and to improve the development of targeted therapy methods. Humans currently have a profound understanding of the induction of cytokine expression and apoptosis. Regulating the expression of various factors inside and outside the cell can affect the active state of HSCs and the function of differentiated tumor and hematopoietic cells, thus producing therapeutic effects [145].

### Resting HSCs and hematopathy

#### Resting HSCs and leukemia

The occurrence of leukemia is related to stress stimuli such as infection and inflammation, which change the surrounding microenvironment and niche of the BM. Resting HSCs can be activated and begin to proliferate, generate hematopoietic cells through multi-directional differentiation, and consequently induce the progression of leukemia [148, 149]. There are abnormal genetic and epigenetic changes that occur during the procession of leukemia [150, 151]. Acute myeloid leukemia (AML) is a malignant tumor of the hematopoietic system characterized by the abnormal proliferation and differentiation of HSCs, which is related to oxidative stress caused by an ROS homeostasis disorder [152, 153]. High ROS levels drive the non-growth factor-dependent proliferation of primitive AML cells [154, 155], leading to DNA damage [156], and this may also lead to a poor prognosis [157]. The ROS levels of CD34 + cells in AML also affect the expression of BCL2. BCL2 mitochondrial regulation can induce therapeutic resistance in leukemia stem cells, which may be related to different therapeutic resistances [158]. HUWE1 is a ubiquitin E3 ligase, that contributes to the proliferation of leukemia cells, affects the differentiation of human HSCs, and may thus provide a new direction for the targeted treatment of leukemia [159, 160]. Other studies have found that CDK2 inactivation can overcome the stagnation of AML cell differentiation and affect the functions of HSCs.

Although there is currently no available selective CDK2 inhibitor, and the AML differentiation caused by inhibiting the expression of CDK2 is not significant, promoting the rapid and effective degradation of CDK2 and inducing the significant differentiation of AML cells still represent a promising method by which to treat leukemia [161].

#### Resting HSCs and myelodysplastic syndrome

Myelodysplastic syndrome (MDS) is a clonal disease characterized by infectious hematopoiesis [162]. Multiple factors regulating the resting state of HSCs are intrinsically related to the occurrence and development of MDS. TET2 is a key regulator of HSCs homeostasis, and somatic mutations in the TET2 gene can occur with both leukemia and MDS. TET2 dysfunction leads to the amplification of HSCs and MPPs in the BM, leading to the accumulation of precancerous clones and induction of disease [61]. Foxm1 is a protein that regulates the cell cycle, and studies have shown that its downregulation or deletion damages the normal functions of HSCs, reduces the survival rate of HSCs after stress stimulation, and leads to hematopoietic abnormalities, and subsequently, MDS [163].

The specific role of resting HSCs in MDS is still unclear, and consequently, further research into the pathogenesis of MDS is required to identify targeted treatment methods and new drug options.

#### Resting HSCs and aplastic anemia

A lack of HSCs can be related to the onset of aplastic anemia (AA), but how the number of resting HSCs changes in AA patients has shown differing results in the research.

FZR1 is a regulatory factor that helps maintain the resting state of HSCs. Chengfang et al. reported on the relationship between FZR1 and severe aplastic anemia (SAA) and found that the expression of FZR1 in HSCs of SAA is downregulated and that insufficient FZR1 will lead to the reduction of resting HSCs and impaired self-renewal ability. The FZR1 deficiency appears to inhibit the ubiquitination of the RUNX1 protein lysine, and this leads to the accumulation of RUNX1 protein and disrupts the resting state of HSCs; consequently, the resting HSCs are reduced in patients with SAA [164]. In addition, Jia et al. detected the expression of Anapc2, a core subunit that promotes the complex/cell cycle body at the late stage of cell division and is critical to the process of cell cycle, in CD34 + HSCs of AA patients. After the induction and knockout of Anapc2, mice quickly developed BM failure, and the number of HSCs in the body rapidly decreased. BrdU labeling showed that CD34 + cells were significantly reduced and that the resting HSCs were rapidly lost, eventually leading to a reduction in whole blood cells [165].

Clinical observations, however, have found that AA patients with effective treatment could recover hematopoietic function without HSCT, and the number of HSCs and peripheral blood cells in the BM increased, suggesting that not all HSCs in AA patients were depleted, or that the HSC damage was reversible [166]. In AA patients, the increase in BM adipocytes inhibits the hematopoietic function of the BM, and its inhibition of lymphoid differentiation may reduce the attack on the hematopoietic system, further protecting the remaining HSCs, helping them maintain their resting state, and preserving the possibility of recovery, which is also consistent with the stress processes in the body [167].

The number of resting HSCs in AA patients is related to disease progression, and inhibiting the consumption of resting HSCs can effectively restore BM hematopoietic function. Therefore, it is particularly important to protect resting HSCs in AA patients, which is expected to result in better clinical efficacy. The occurrence of various hematopathies is closely related to resting HSCs, and current evidence indicates that the treatment of diseases based on the functional activity of HSCs should be investigated further.

### Resting HSCs and tumors

The abnormal functions of HSCs are also related to the occurrence of various tumors. Cancer stem cells (CSCs) are a subset of HSCs located in tumors that can self-renew and differentiate. CSCs are associated with aggressive tumor phenotypes and drug resistance [168]. CSCs have also been detected and isolated from many solid cancers, such as lung, liver, ovarian, and pancreatic cancers, and changes in the hematopoietic microenvironments of tumors can change the fate of the CSCs [94], leading to tumor occurrence.

Trilaciclib is a small molecule, a short-acting CDK4/6 inhibitor with BM protection and potential anti-tumor effects, which can induce HSC proliferation in the BM to temporarily and reversibly stagnate the G1 phase, protect them from damage during chemotherapy, help them to recover more quickly from hematopoiesis, and enhance anti-tumor immunity [169, 170]. Lowell et al. conducted an experiment involving 61 patients with small cell lung cancer, and the main endpoint of the study was the occurrence and duration of severe neutropenia (DSN) in the first cycle. The results showed that the administration of trilaciclib before chemotherapy reduced the incidence and duration of DSN in the first cycle, patients had fewer hematological adverse events (≥ grade 3), and overall safety was improved [171]. Another experiment involving 122 patients with extensive small cell lung cancer reached a similar conclusion, confirming the BM protection function of trilaciclib [172]. Studying the regulatory mechanisms that affect the self-renewal of HSCs is an important way to identify tumor-specific targeted drugs; however, the relationship between factors that affect the functions of HSCs and CSCs is still unclear, which suggests an important direction for future research.

### Resting HSCs and other systemic diseases

HSCs are common precursors of all blood cells in the body, are distributed in multiple systems, and participate in the development of various diseases. In addition to hematopathy and solid tumors, the regulation of HSCs is also related to autoimmune diseases, myocardial damage, and nervous system damage.

#### Resting HSCs and autoimmune diseases

An animal model study found that abnormal HSCs may also induce autoimmune diseases (AIDs) [173], and transplanted HSCs from AID mice into normal mice induced AIDs in recipient mice, suggesting that AIDs are rooted in the functional defects of HSCs and can thus be transferred via HSCs [174]. Autologous hematopoietic stem cell transplantation (auto-HSCT) receives islet tissues from donors, resulting in tolerance to self-islet cells [175]. Many patients with type I diabetes (T1DM) can stop insulin through auto-HSCT [176] and prevent and treat pancreatic islet inflammation in T1DM mice [177], which can also transform the lineage, induce islet cell regeneration, and improve the overall cure rate [178]. Compared with mice without HSCT, T1DM mice treated with HSCT have higher serum insulin levels, lower lymphocyte infiltration levels, and a lower percentage of CD8 + T cells, the survival rate of mice was found to be higher, which confirms the role of HSCT in improving islet cell tolerance, effectively treating T1DM, and improving hematopoietic function [179]. These findings indicate that HSC dysfunction is closely related to the occurrence and development of T1DM, and HSCT treatment based on this mechanism is an important clinical strategy [180].

Auto-HSCT is the only proven long-term effective method to treat systemic sclerosis, and it can eliminate the pathogenic autoreactive immune cells of patients, restore the HSCs in the body, and enable re-entry into immune homeostasis [181].

Abnormalities in immune cells in systemic lupus erythematosus (SLE) can also be traced to HSCs; the proliferation and differentiation of CD34 + cells in SLE patients are enhanced, the transcription of cytokines and chemokines is activated, and the activation of resting HSCs may aggravate the inflammation and attack risk of SLE [182].

The occurrence of rheumatoid arthritis is also related to abnormalities in BM lineage immunity and HSCs, with premature aging of the T cells, and its mechanism is mostly related to telomere loss and DNA repair defects [183, 184]. The occurrence of multiple AIDs is inseparable from HSCs and is the focus of ongoing clinical treatments.

#### Resting HSCs and myocardial injury

HSCs can circulate to the injured site after cardiac injury to help myocardial repair and regeneration, and the abnormal function of HSCs is related to an increase in the incidence of cardiovascular diseases. Studies have shown that BM can induce emergency hematopoietic regulation after ischemic injury [185], and the mechanism of regulating HSC quiescence can also affect cardiac function. For example, mutation of the epigenetic regulator TET2 can lead to cardiac injury, affect cardiac repair function, and even heart failure [186]. Meis1 is a transcription factor that positively keeps HSCs at rest and plays an important role in heart regeneration and stem cell function. Knockout of Meis1 in myocardial cells activates resting HSCs leads to HSC expansion and causes myocardial cell proliferation [187]. CD34 + cells are multifunctional HSCs, that can be used to treat ischemic myocardial injury [188], and have the ability to promote the regeneration of myocardial cells [189]. The effectiveness of CD34 + cell implantation in the treatment of myocardial infarction (MI) has been confirmed [190]. The lung is also a repository of HSCs, in which HSCs can be located in the damaged heart to promote endogenous repair, which has been proven safe and effective in a mouse model of MI [191]. HSC therapy is considered a promising cardiac repair strategy, but the current clinical application of such methods is limited, and the adverse reactions and repair mechanisms of HSCT are elusive. Therefore, studies on the role of HSCs in heart damage are expected to lead to better repair of myocardial injury.

#### Resting HSCs and nerve injury

Targeted clinical treatment of nervous system injuries is very complex. Studies have shown that HSCs can affect peripheral auditory nerve (AN) cells. After AN injury, the number of HSC-differentiated downstream cells increases, and they participate in the remodeling of AN [192]. Apoptosis damage to the sympathetic nervous system (SNS) leads to a significant reduction in the number of HSCs in neuropeptide Y-deficient mice, and impaired BM regeneration function, which can promote neuroprotection and restore BM dysfunction caused by SNS damage by regulating the BM microenvironment [193]. Clinical anticancer chemotherapy drugs can cause severe sensory neuropathy [194], which can be treated by HSC-targeted therapy. Cord blood CD133 + HSCs with self-renewal and neural lineage differentiation ability can play a role in differentiating into motor neuron-like cells in vitro [195]. Clinical reports have confirmed the benefits of HSC therapy in protecting the nervous system from damage [196].

Nerve injury-related diseases seriously affect the lives of patients, and HSC therapy has become a focus of scientists as a good strategy to recover lost cells.

## Conclusions

HSCs are the “source” of various hematopoietic cells, and the maintenance of normal hematopoietic function depends on their self-renewal, replication, and multi-lineage differentiation. When the body is in a normal steady state, most HSCs are in a resting state similar to “dormancy”. However, after encountering a stress stimulus such as an infection, DNA damage or tumor development, the resting HSCs can be re-activated. The resting state is key for HSCs to ensure their long-term survival, normal function, and the maintenance of hematopoiesis and regeneration for life. Many regulatory mechanisms can affect the resting state of HSCs, leading to abnormal hematopoietic function and the emergence of multi-system diseases. Currently, HSCT remains the mainstay of treatment for many hematologic diseases, malignancies, and genetic disorders, and the ability to replenish mature blood cell lineages and preserve the survival of quiescent HSCs is key for the clinical development of HSCT. This review indicates that genetic, epigenetic, microenvironmental, and homing HSC modulations are required for HSC functional activity, and these data could help identify important directions for new clinical therapies. Many studies have confirmed the involvement of HSCs in the clinical treatment of multiple systemic diseases, and although the reinfusion of pluripotent stem cells to treat diseases is more controversial, this remains an important strategy for clinical treatments, considering the limited availability of HSCT. The dynamic regulation between resting HSCs and multiple mechanisms may be the focus of specific targeted therapies in the future.

However, research on the regulatory network of resting HSCs is currently limited, and the existing ideas are at a theoretical level. Furthermore, specific changes in the immune system after the activation of resting HSCs and the relationship between the resting state of HSCs and the prognosis of disease development are not yet fully understood. There have been few clinical verifications involving resting HSCs therapy, and the sorting and activation of resting HSCs in the human body remains unclear. The interactions between resting HSCs and other parts of the hematopoietic system should be investigated to design a new clinical strategies for the treatment of disease. However, to achieve this, we must increase relevant clinical research. Targeted therapy for hematologic and other systemic diseases through the in vivo and in vitro induction of directed mobilization and differentiation of quiescent hematopoietic stem cells is also a possible future strategy.

## Data Availability

Not applicable.

## Abbreviations

HSCs: Hematopoietic stem cells
BM: Bone marrow
LT-HSCs: Long-term hematopoietic stem cells
MPPs: Multipotent progenitor cells
CDK: Cyclin-dependent kinase
CKI: Cyclin-dependent kinase inhibitor
CIP/KIP: Cytokine-induced protein/kinase
LAP2α: Layer-related polypeptide 2alpha
APC/C: Anaphase-promoting complex/cyclite
hASCs: Human adipose stem cells
DNMT3: DNA methyltransferase 3
DDR: DNA damage response
ROS: Reactive oxygen species
LTC-IC: Long-term culture of starting cells
OGT: O-linked N-acetylglucosamine transferase
HIF-1α: Hypoxia-inducible factor-1 α
LepR: Leptin receptor
VLA-4: Very late antigen 4
CAM: Cell adhesion molecule
AML: Acute myeloid leukemia
MDS: Myelodysplastic syndrome
AA: Aplastic anemia
SAA: Severe aplastic anemia
CSCs: Cancer stem cells
DSN: Duration of severe neutropenia
HSCT: Hematopoietic stem cell transplantation
T1DM: Type I diabetes
SLE: Systemic lupus erythematosus
MI: Myocardial infarction
SNS: Sympathetic nervous system
AN: Auditory nerve
AIDs: Autoimmune diseases
